# Ultrastructural Analysis of In Vitro Glycated Engineered Skin

**DOI:** 10.1111/jocd.70747

**Published:** 2026-02-19

**Authors:** Kimberly Denman, Vighter Iberi, Yuri Roiter, Madison Ammon, Bin Fang, Ravi Ranatunga, Gunjan Agarwal

**Affiliations:** ^1^ Department of Biomedical Engineering The Ohio State University Columbus Ohio USA; ^2^ Procter and Gamble Company Mason Ohio USA; ^3^ Biophysics Program The Ohio State University Columbus Ohio USA; ^4^ Department of Mechanical Engineering The Ohio State University Columbus Ohio USA

**Keywords:** atomic force microscopy, glycation, mechanics, scanning transmission electron microscopy, skin

## Abstract

**Background:**

Aging of the human skin results in undesirable physical properties such as wrinkles, yellowing, and loss of luster. Advanced glycation end‐products (AGEs) are understood to be one of the primary causes for these effects as assessed from studies on human skin subjected to in vivo or in vitro mediated glycation. Models to recapitulate the effect of skin‐glycation in vitro are an active area of interest to understand and mitigate these effects. In this regard, engineered skin models have utilized pre‐glycated collagen to assemble a glycated skin layer. However, this method is time‐consuming and can suffer from variability across samples. The objective of this study was to examine how in vitro glycation of an engineered skin model affects its material properties.

**Materials and Methods:**

In this study we used a pre‐engineered skin model (MatTek EpiDerm) and subjected it to in vitro glycation using glyceraldehyde. The changes in luminosity of the samples were characterized 48 h post‐glycation. Immunohistochemistry using an anti‐AGE antibody was performed to verify glycation of the samples. Scanning transmission electron microscopy (STEM) and atomic force microscopy (AFM) were used to assess the ultrastructure of the samples and evaluate their surface roughness, adhesion, and mechanical properties.

**Results:**

Our results show that upon glycation the engineered skin had reduced luster, increased yellowing, disruption in cell and matrix morphology, increased roughness, and modulus. These effects are similar to those previously reported in both natural and engineered skin.

**Conclusions:**

Overall, the approach presented here serves as a quick and easy method to recapitulate the glycation induced effects in the model skin and can serve as a platform to evaluate the effect of skin‐care products on mitigating these effects.

## 1 Introduction

The skin is the largest organ in the human body and is the first line of defense from many illnesses. In addition to keeping individuals healthy, skin plays an important role in confidence and appearance. It is well known that with aging, skin loses its luster and is accompanied by changes in dermal thickness, yellowing and wrinkles [1]. These age‐related changes are understood to be in part due to the accelerated formation of multiple advanced glycation end‐products (AGEs). AGEs accumulate during aging because the normal biological processes responsible for removing them are compromised [2]. In addition, other factors such as dietary sugar intake, diabetes and UV exposure can exacerbate AGE accumulation [2].

Studies on natural skin have yielded valuable insights into the undesired outcomes due to accumulation of AGEs or in vitro glycation. The accumulation of AGEs with aging is reported to increase the stiffness of skin and impair its elasticity [3]. Skin from aged subjects had increased fragmentation and disorganization of collagen fiber bundles as compared to the young skin, resulting in higher surface roughness of skin tissue sections when analyzed by atomic force microscopy (AFM) [4]. In another study, ultrastructural analysis by transmission electron microscopy (TEM) revealed the presence of an amorphous matrix amidst the collagen fibrils in the dermis of diabetic patients, and X‐ray diffraction noted loss of the characteristic D‐periodicity of collagen fibrils [5]. In vitro treatment of human skin has been shown to mimic these in vivo effects such as yellowing of epidermal punch biopsies upon treatment with glyceraldehyde (GA) [6]. In vitro ribose mediated glycation of young skin resulted in increased stiffness and hardness as compared to nascent young skin [4].

The use of engineered skin to mimic these age‐related changes is an active area of interest to better understand the underlying changes in cell and matrix environment and devise strategies to combat these adverse changes by use of skin care products. For instance, in vitro glycation of innervated tissue‐engineered skin models by treatment with glyoxal over several weeks resulted in epidermal differentiation defects, which could be prevented by addition of an anti‐glycation compound [7]. Other studies have used reagents such as, ribose, sodium glyoxylate, or Nepsilon‐(carboxymethyl‐lysine: CML) to glycate collagen in vitro and utilize it to assemble reconstructed human skin model [8, 9, 10]. The glycated skin model used in these studies appeared yellowish compared to non‐glycated skin and revealed aggregation of collagen [8]. Furthermore, the use of glycated collagen matrix in the dermal compartment impacted the epidermal layer morphology. While these models have been helpful to mimic the glycation in aging skin, the layer‐by‐layer assembly of engineered skin is time intensive and can suffer from sample variations.

In this study we subjected a pre‐assembled engineered skin model to in vitro glycation and analyzed changes in its luminosity, ultrastructure, and nanomechanical properties. We elucidate how the resulting changes compare to those reported in naturally aged skin as well as in reconstituted skin models which utilize glycated collagen.

## 2 Methods

### 2.1 Tissue Samples and Treatment

A commercially available engineered skin model, namely MatTek EpiDermFT (BICO) was utilized for in vitro glycation studies. Following a similar protocol as stated in Fang et al. [11] the model skin samples (12 mm diameter) were cultured in Dulbecco's Modified Eagle's Medium, (DMEM) (from MatTek) for 24 to 48 h at 37°C with 5% CO_2_ in accordance with manufacture guidelines. To induce glycation, both the model skin samples were treated with 12.5 and 2.5 mM glyceraldehyde (GA) in EpiDerm media (BICO) for 48 h. Following this treatment, the samples were rinsed and processed for the following analysis.

### 2.2 Color Analysis

The skin samples before and after treatment with GA were washed (three times) in phosphate buffer saline (PBS) and imaged using a spectrophotometer (MetaView VS3200, X‐Rite) to acquire L* a* b* spectra values with *n* = 3 scans at a diameter of 6 mm taken from different regions of the tissue. L* value denotes lightness (brightness), a* the redness, and b* the yellowness. The change in color (ΔE*) of each sample before and after glycation was calculated using the following equation, where the subscripts 1 and 2 denote before and after glycation treatment:

$$∆E^{*}=\sqrt{{\left(L_{2}-L_{1}\right)}^{2}+{\left(a_{2}-a_{1}\right)}^{2}+{\left(b_{2}-b_{1}\right)}^{2}}$$

Another set of skin samples was similarly treated and thereafter cut into four segments for microscopy analysis. Two of the segments were embedded in optimal cutting temperature (OCT) media, flash frozen in liquid nitrogen and stored at −80°C for cryo‐sectioning. Another two segments were fixed in glutaraldehyde for electron microscopy.

### 2.3 Immunohistochemistry

Cryosections (5 μm thickness) of OCT embedded skin tissue were collected on glass slides and prepared as previously described [12, 13]. Sections were fixed with 2% paraformaldehyde (PFA) in PBS (5 min at RT) and then washed with PBS (3 × 10 min at RT). Sections were then permeabilized with 0.2% Triton X‐100 in PBS (15 min at RT) and incubated with blocking buffer (5% Normal Donkey Serum, 0.1% triton in PBS for 2 h at RT). Samples were labeled with primary antibodies (overnight at 4°C), washed in PBS (3 × 5 min at RT), labeled with secondary antibodies (2 h at RT), and washed again in PBS (3 × 5 min at RT) with the last wash including a nuclear stain (1:10 000; Hoescht 33342 nucleic acid stain, ThermoFisher Scientific). Slides were mounted in Prolong Glass (Invitrogen by ThermoFisher Scientific) and allowed to cure for 48 h at RT before imaging. Anti‐AGEs mouse monoclonal antibodies (0.25 mg/mL; Trans Genic Inc) and goat anti‐mouse secondary antibodies conjugated to Alexa 647 were used (1:8000; ThermoFisher Scientific).

Confocal imaging was performed with a Nikon AX/R laser‐scanning confocal microscope equipped with solid‐state lasers (405, 488 and 647 nm, 30 mW each), a 63×/1.4NA oil‐immersion objective, a spectral detection module comprised of 2 GaAsP detectors and 2 high‐sensitivity photomultiplier tube detectors, a cutting‐edge high‐definition confocal scanner, and a high‐speed piezo z‐drive. Nuclear and AGE staining were analyzed using excitation wavelengths of 405 and 647 nm respectively, while autofluorescence was ascertained at an excitation of 488 nm. Images were analyzed using ImageJ by first subtracting the background and then finding the mean intensity.

### 2.4 Scanning Transmission Electron Microscopy (STEM)

A segment of the skin samples was fixed in 3% glutaraldehyde overnight at 4°C, washed twice in PBS for 10 min, and then fixed in 1% osmium tetroxide for 1 h. Samples were then washed twice in PBS for 10 min, followed with en bloc staining with 2% uranyl acetate for 1 h. Dehydration was performed using a graded acetone series followed by embedding in low‐viscosity Spurr's epoxy resin. The embedded samples were sectioned to ~95 nm thickness using a *Leica* UC6 ultramicrotome while ensuring the layers from stratum corneum to the dermis were in view. Sections were transferred to formvar coated mesh copper grid and post stained with lead citrate for 5 min. The ultrastructure of the skin sections was observed on a *Hitachi S‐5200* scanning/transmission electron microscope (STEM) with an accelerating voltage of 30 kV.

### 2.5 Atomic Force Microscopy (AFM)

OCT embedded samples were cut to 3 μm thick sections (comprising both the epidermal and dermal layers) using a cryostat and placed on pieces of silicon wafers. The silicon wafers were pre‐cleaned with air plasma (Harrick Plasma, 18 W) and then treated with a 2% (vol) diethylene‐triaminetrimethoxysilane (DETAS, Gelest) solution in methylethylketone overnight and finally rinsed with ethanol and dried. The tissue sections mounted on silicon wafers were washed twice with PBS and twice with distilled water and then air dried for 1 h in a chemical safety hood.

Samples were examined on a *Bruker Multimode 8* AFM with ScanAsyst‐Air (Bruker) probes (nominal 0.4 N/m spring constant, nominal frequency of 70 kHz) in ambient air. Deflection sensitivity was measured on sapphire, and the spring constant was evaluated using the thermal tune method. The radius of curvature was evaluated to be approximately 30 nm. A top‐view camera was used for placement of the probe in the stratum corneum, epidermis, and dermis regions. Peak Force (PF) Quantitative Nano Mechanics (QNM) mode in air was used to acquire mechanical and topographical data at a PF Setpoint of 30 nN. Height, PF‐error, DMT modulus, and adhesion maps were acquired for each region using scan sizes of 1, 2, 5, and 10 μm. The average surface roughness, DMT modulus, and adhesion were ascertained by analyzing 1 × 1 μm areas (*n* ≥ 100 scans), using MountainsSPIP (Digital Surf).

## 3 Statistical Analysis

Two‐tailed, unpaired *t*‐tests with equal variances were used for comparison between treatments at each time point using RStudio with R version 4.4.1 [14, 15, 16, 17]. Significance was determined by *p* ≤ 0.05.

## 4 Results

### 4.1 Effect of Glycation on the Color of Engineered Skin

**FIGURE 1:**
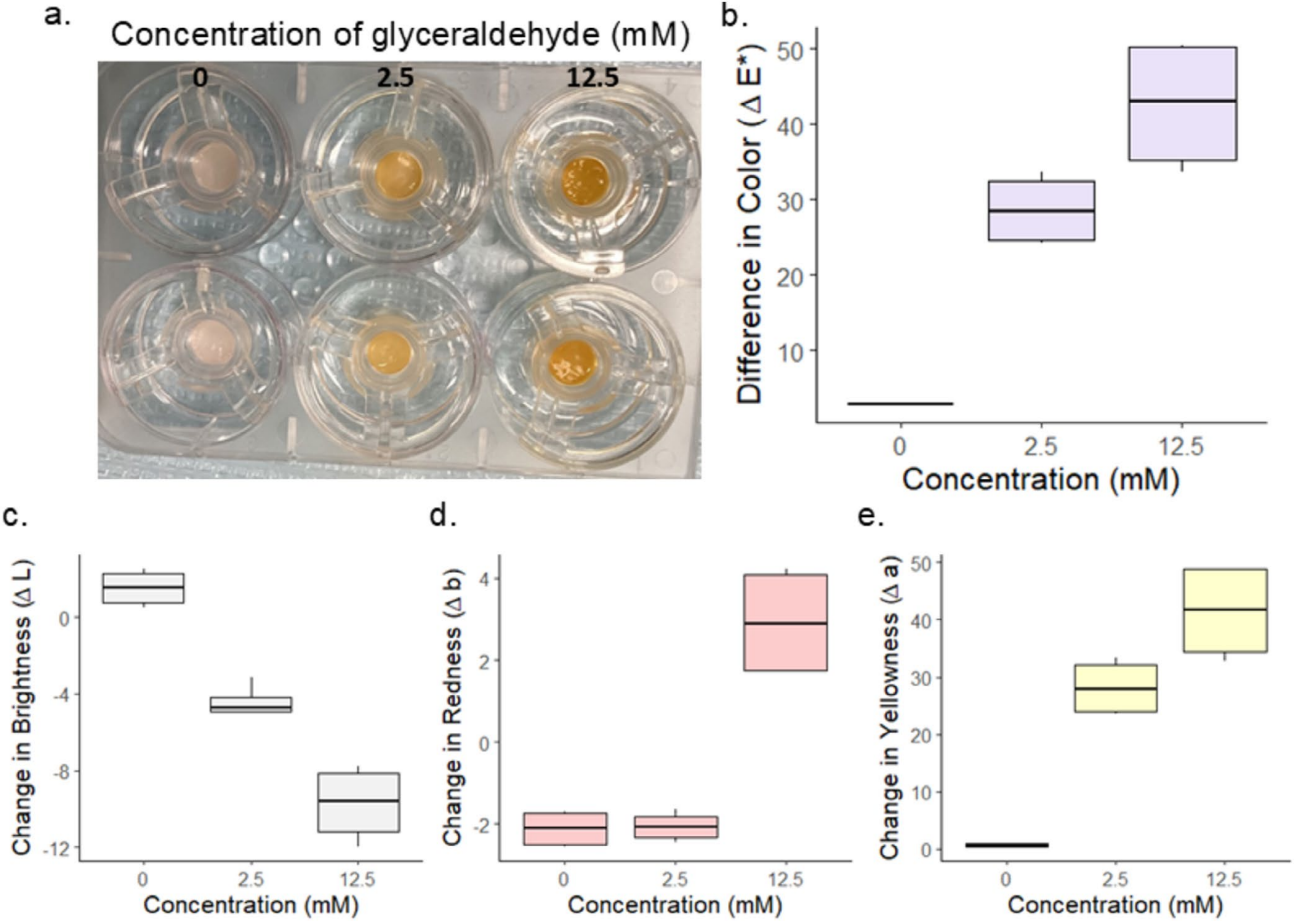
Optical appearance of in vitro treated skin model. Image of model skin taken 48 h after exposure to glyceraldehyde at 0, 2.5, and 12.5 mM concentrations (a). Overall color change, Δ*E*, after 48 h (b), change in brightness (c), change in redness (d), and change in yellowness (e) is shown for each concentration.

The *L*.*a*.*b* values of the engineered skin punches were measured before subjecting them to 0, 2.5 or 12.5 mM GA. After exposure to GA for 48 h, there was a distinct change in the appearance of the samples (Figure 1a). The color change (Δ*E*) exhibited a concentration dependent increase (Figure 1b). To account for this, we also analyzed the individual parameters L*, a*, and b*. Upon glycation, a decrease in lightness (L*) was observed as compared to controls (Figure 1c). The change in redness (Δ*a*) was only apparent at the higher GA concentration (Figure 1d), while an increase in yellowness (Δ*b*) was present in a concentration dependent manner (Figure 1e). Using a two tailed paired *t*‐test the *p* values for the 2.5 mM and 12.5 mM glycated samples were 0.024, and 0.007 respectively.

### 4.2 Verification of AGE Proteins in GA‐Treated Engineered Skin

**FIGURE 2:**
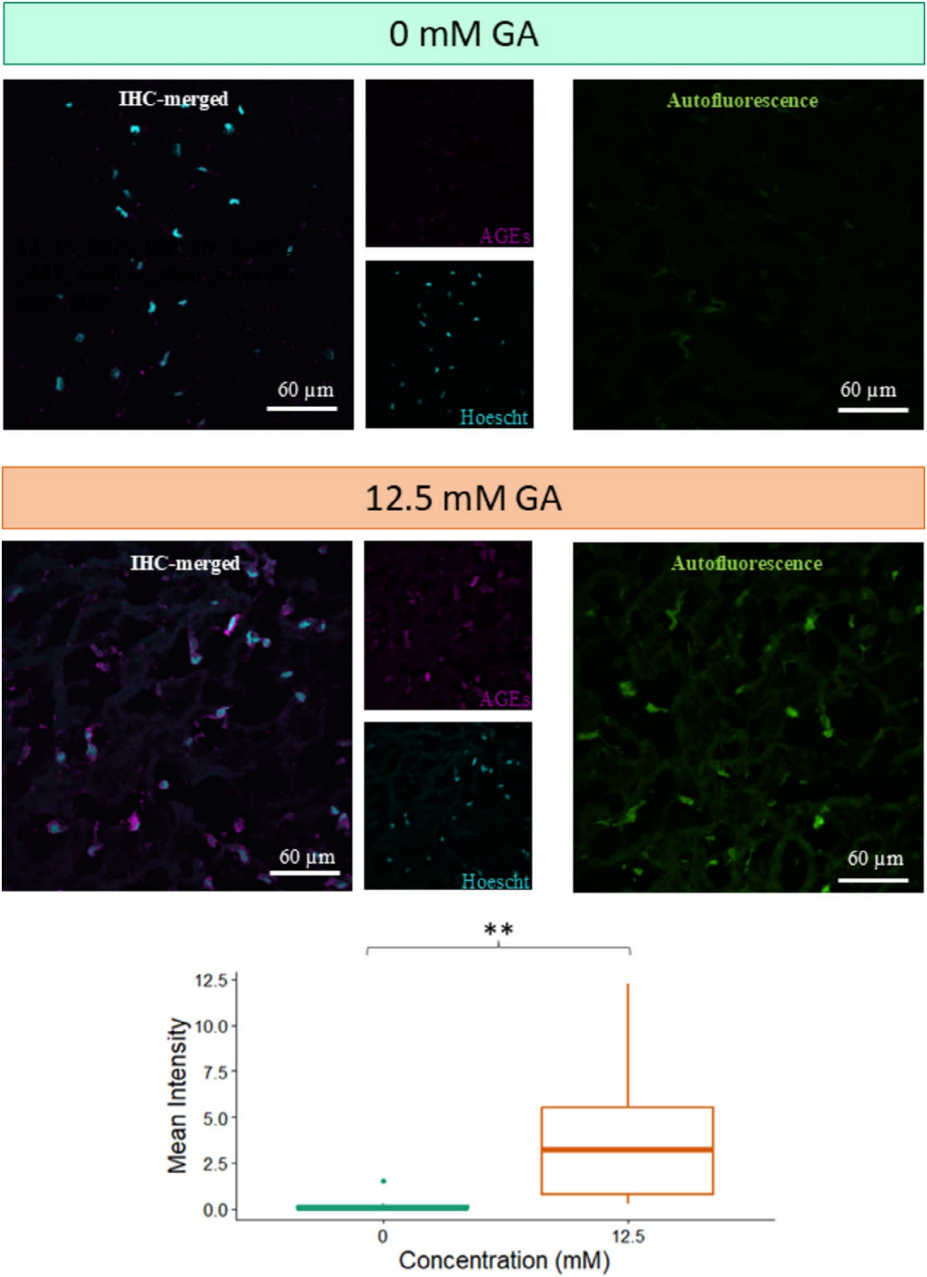
Histological analysis of model skin upon GA treatment. Representative images of immuno‐histological (IHC) staining and autofluorescence images of 0 and 12.5 mM GA‐treated model skin samples (as indicated). A visible increase in AGE‐staining (magenta) around the cell nuclei (cyan) is noted in the 12.5 mM for AGE proteins. There is also a visible increase in autofluorescence of the samples treated with 12.5 mM GA. The mean intensity per pixel of the AGE‐staining showed a significant increase in the AGE signal for 12.5 mM treated samples in IHC images (**p < 0.01).

Immunohistochemistry was performed on the samples 48 h post exposure to 0, 2.5, or 12.5 mM GA to evaluate the presence of AGE‐modified proteins in the skin model. Visually, there was an increase in the amount of AGE signal in the 12.5 mM GA group in comparison to the 0 mM control group (Figure 2). To quantify this difference, mean intensity per pixel was calculated for each image using ImageJ, confirming the increased presence of AGE signal in the 12.5 mM GA group compared to the 0 mM GA group. This effect was deemed statistically significant through using a two‐tailed paired test with a *p* value of 0.005. Additionally, it was observed that the 12.5 and 2.5 mM GA‐treated samples had an increase in autofluorescence in comparison to the 0 mM samples. Together, the statistically significant increase in pixel intensity, corresponding to increased AGE signal, as well as the increase in autofluorescence, in the 12.5 mM GA groups compared to the 0 mM GA, confirmed the presence of glycation.

### 4.3 Effect of Glycation on the Ultrastructure of Engineered Skin

**FIGURE 3:**
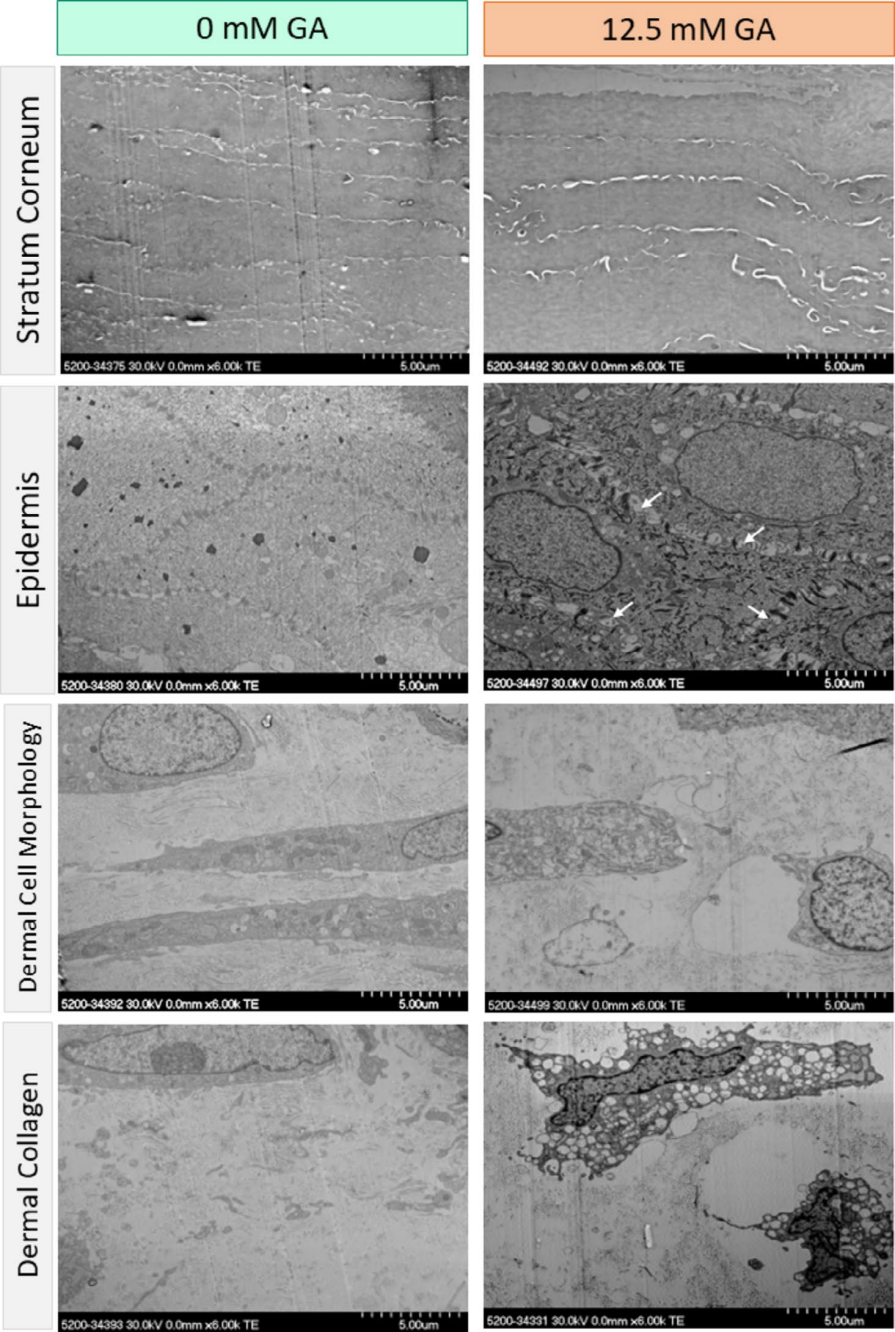
Scanning transmission electron microscopy (STEM) of control and in vitro treated model skin. Representative images of stratum corneum, epidermis, dermal cell, and dermal collagen of control and 12.5 glycated in vitro skin models. No morphological differences were observed in the stratum corneum. Cell–cell junctions in the epidermis exhibited increased gapping upon GA treatment (white arrows). Changes in cell shape and collagen fiber distribution were observed after GA treatment in the dermal layer.

The impact of glycation on the ultrastructure of the in vitro engineered skin model was explored with scanning transmission electron microscopy (STEM). There was no marked change in the layers of the stratum corneum of the engineered skin model. The epidermal layer showed increased separation around the desmosomes after glycation (Figure 3). Furthermore, at the epidermal‐dermal junction, the non‐glycated sample showed a smooth transition between the two layers. However, in the glycated sample there was some gapping. In the dermis of the glycated sample, there was alteration in cell morphology as well as how the collagen was dispersed. The untreated dermis was comprised largely of elongated cells, while GA treatment led to shriveling of cells. The distribution of collagen fibrils also transitioned from a uniform to a more heterogeneous distribution in the GA‐treated skin.

### 4.4 Effect of Glycation on Nanomechanical Mapping of Engineered Skin

**FIGURE 4:**
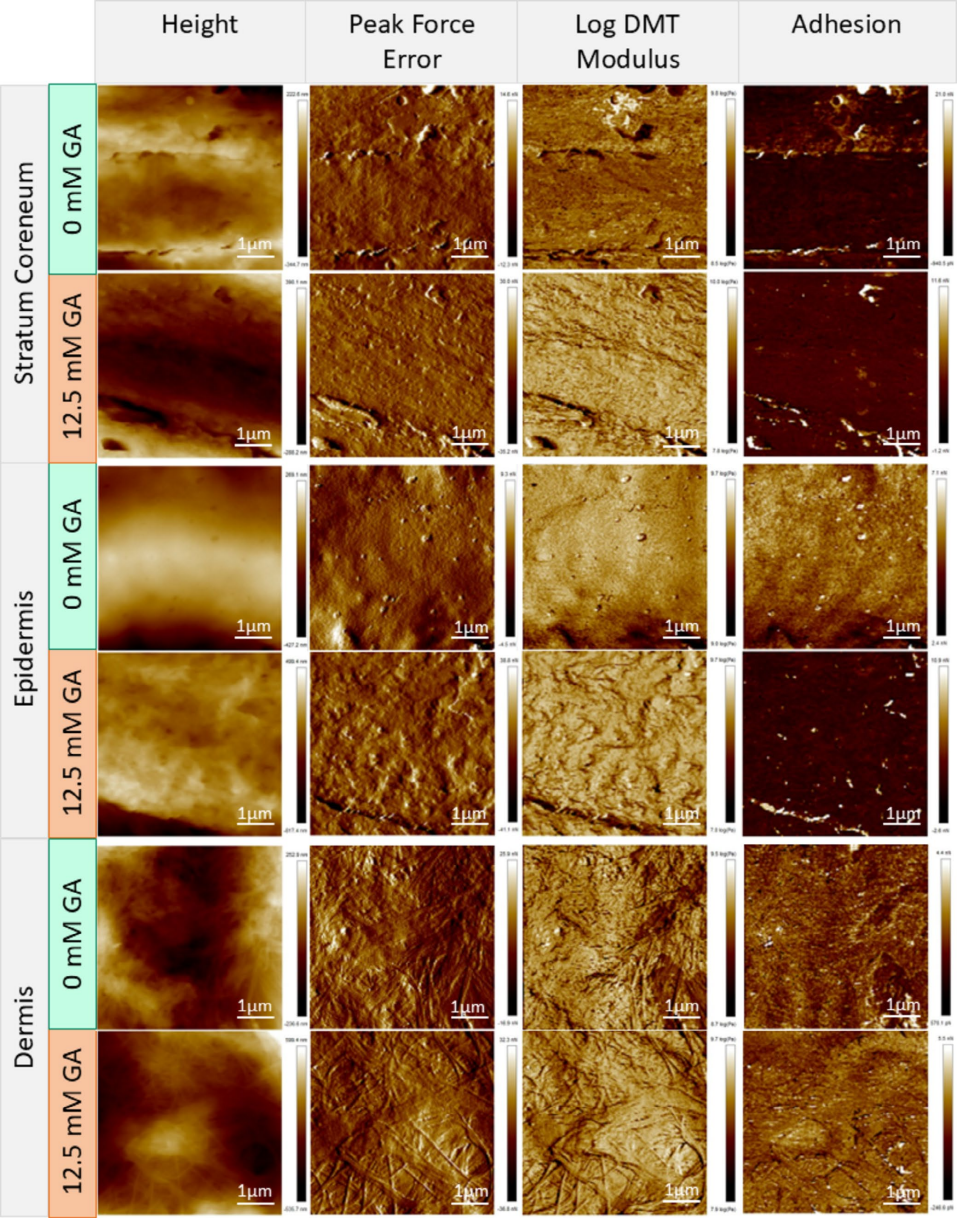
Atomic force microscopy (AFM) analysis of the in vitro treated model skin. Representative images of stratum corneum, epidermis, and the dermis at 0 and 12.5 mM as indicated. The AFM topography (height) image and the corresponding peak force error image, natural log of DMT modulus, and adhesion maps from the same region are presented alongside.

**FIGURE 5:**
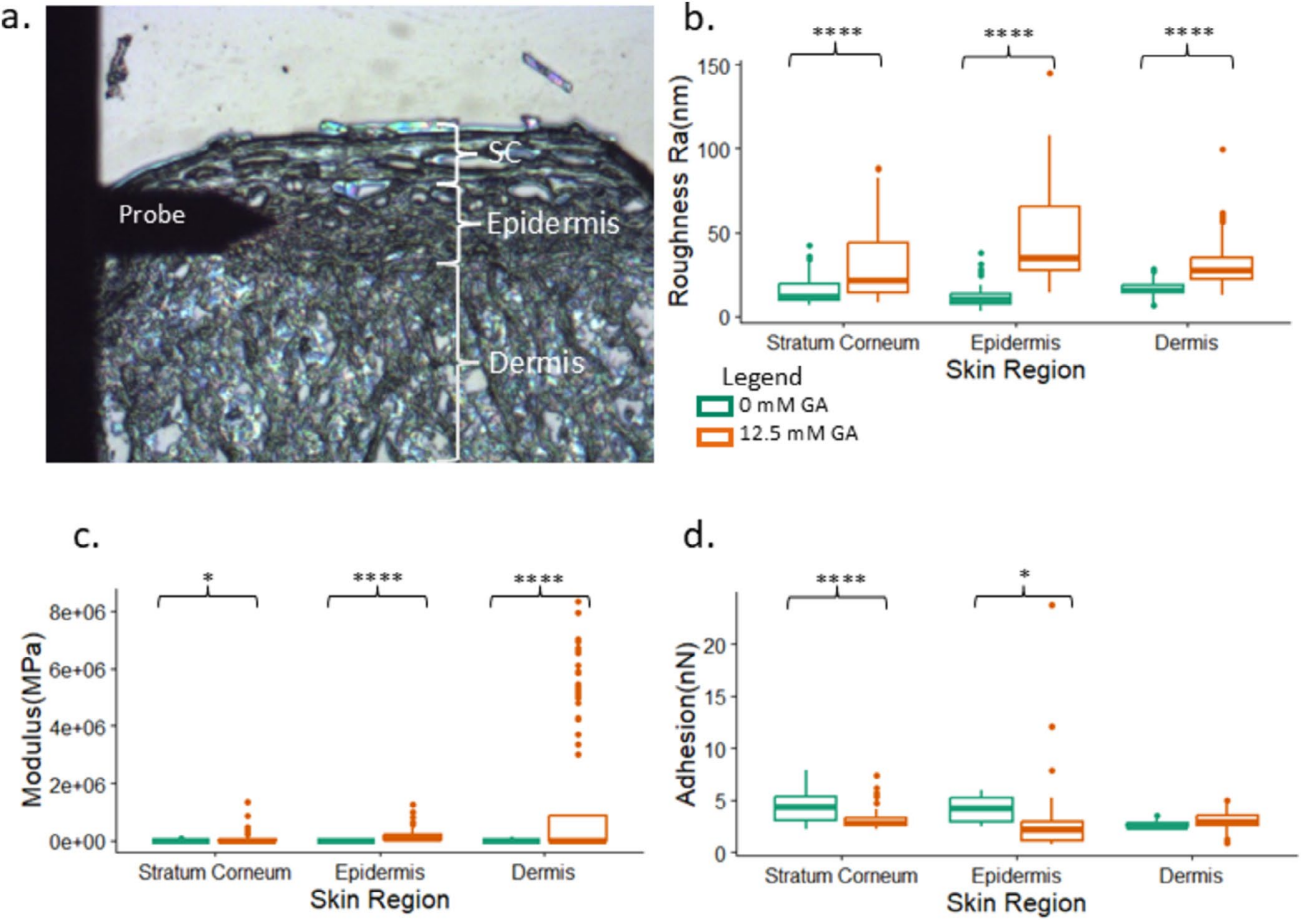
Topographical and mechanical data from AFM analysis of model skin. Top‐down view from MultiMode 8 AFM showing all three layers present in the MatTek EpiDermFT model (a). Comparison of the roughness of the control and glycated in vitro skin model (b). Log of DMT Modulus comparison of control and glycated in vitro skin model (c). Adhesion of control and glycated in vitro skin model (d). *p < 0.05, **p < 0.01, ***p < 0.001, ****p < 0.0001.

To assess the change in mechanical properties and topography of the different skin layers, atomic force microscopy (AFM) was utilized. The top‐down camera on the AFM equipment was used to visualize and determine each layer of the MatTek EpidermFT skin model (Figure 5a). Height, peak force error (PFE), log DMT modulus, and adhesion images are shown for each layer of the skin from control and glycated samples (Figure 4). For all skin layers, Rq, roughness from height measurements, was significantly higher after glycation (Figure 5b). The mean DMT modulus values were significantly increased in all the layers (Figure 5c). In addition, the glycated samples had a much wider spread of modulus values (Figure 5c). Due to the complexity of the sample and its dynamic effect on probe radius, the DMT modulus should be considered relative for all samples. We also analyzed the tip‐sample adhesion from the adhesion maps acquired as part of the PF‐QNM mode. Adhesion varied across the layer of the skin that was analyzed. Only the stratum corneum and epidermis showed a significant decrease post‐glycation, whereas the dermis showed no difference (Figure 5d).

## 5 Discussion

This study aimed to characterize changes in a model of engineered skin after in vitro glycation. The GA‐treated samples showed increased AGE‐staining, verifying in vitro glycation. The model skin showed a statistically significant increase in b* values 48 h post glycation for both concentrations of GA. These results are consistent with a previous study where treatment of human skin biopsies with GA resulted in increased b* [6]. In natural skin, a correlation between yellowness and perceived dullness has been reported for both young [18] and aged skin [19]. We did not observe a significant change in redness upon GA treatment. These results are consistent with previous reports, where AGE accumulation has been positively correlated with skin yellowness but little [18] to no [19] correlation with redness was reported.

Electron microscopy showcased distinct changes in the 12.5 mM GA glycated samples of the engineered skin model. The glycated samples showed no apparent change in the stratum corneum, while the epidermal layer showed increased spacing between cells. Our results are consistent with previous studies, which have reported that glycation results in disruption of keratinocyte cells, vacuolation of the cytoplasm, and damage to the skin barrier function [20]. In addition, the permeability of the stratum corneum and the dermal‐epidermal junction has been found to be altered post glycation [21].

Our AFM analysis revealed increased surface roughness and DMT modulus in all glycated samples compared to their non‐glycated counterparts in each layer of the skin. Our results are consistent with previous studies on the impact of glycation, which report increased stiffness and roughness across a variety of tissues [22, 23, 24]. Previous AFM studies also revealed that ribose treatment of human skin biopsies increased the stiffness and hardness in both the reticular and papillary dermis [4]. Analysis of dermal collagen fibers in natural skin (from hip and forearm) also showed increased stiffness in aged individuals [4, 25]. Our AFM results also show a change in the adhesive properties of the epidermis and SC upon GA treatment, with no significant change in the dermis. We postulate that the increase in adhesion in the SC and epidermis could be due to changes to the cells and lipid distribution in these layers upon GA treatment that could cause changes in probe‐sample adhesion.

Taken together, we show how in vitro glycation of engineered model skin can mimic the effects of glycation in natural skin. We acknowledge the limitations of our study, including sample size and the fact that the skin mimic is not an exact replica of human skin and fails to account for alterations in skin tones. Nevertheless, our protocol can serve as a quick and easy method to utilize engineered skin to mimic the effects of glycation in vitro and test agents to mitigate this effect.

## Author Contributions

K.D. and M.A. contributed to the acquisition of data, analysis and interpretation, and drafted the manuscript. V.I., Y.R., B.F., and R.R. contributed to conception and design and interpretation of data. G.A. contributed to analysis and interpretation of data. All authors contributed to revising the manuscript for intellectual content.

## Funding

This work was supported by the National Science Foundation (2000469).

## Ethics Statement

The authors have nothing to report.

## Conflicts of Interest

The authors declare no conflicts of interest.

## Data Availability

The data that support the findings of this study are available upon request from the corresponding author.
